# Intraoperative Cycling Pressure Variation in the Treatment of Central Retinal Artery Occlusion

**DOI:** 10.1155/2021/6649657

**Published:** 2021-01-13

**Authors:** A. Altun

**Affiliations:** Department of Ophthalmology, Bahcesehir University, Istanbul, Turkey

## Abstract

A 45-year-old male presented to the clinic of ophthalmology with central retinal artery occlusion (CRAO). There was no response to medical treatment, ocular massage, and anterior chamber paracentesis. CRAO was resolved by pars plana vitrectomy and intraoperative cycling pressure variation. The best-corrected visual acuity improved to 20/100 on the first day and to 20/20 on the first month, postoperatively.

## 1. Introduction

Central retinal artery occlusion (CRAO) is an ophthalmological emergency because the sudden and catastrophic vision loss might be permanent. Perfusion of the retinal ganglion cell layer is provided by end-artery circulation of the retinal artery [1]. In CRAO, infarction develops in the inner retina and retinal ganglion cells [2]. Providing rapid and early reperfusion is vital in CRAO.

Many medical and surgical treatment methods have been defined for the management of CRAO. Digital ocular massage [3], acetazolamide [4], intravenous mannitol [5], oral pentoxifylline [6], inhalation of 10% carbogen [7], sublingual isosorbide dinitrate [8], intravenous methylprednisolone [9], intravenous or intra-arterial recombinant tissue plasminogen activator [2], hyperbaric oxygen [10], anterior chamber paracentesis [11], and Nd:YAG (neodymium-doped yttrium aluminum garnet) laser embolectomy/embolysis [12] are the most known alternatives.

Technological advances in microsurgery have recently led to the more frequent application of vitreoretinal surgical interventions in the management of CRAO [13]. In this paper, we aimed to present a case with CRAO resolved by pars plana vitrectomy (PPV) and intraoperative cycling pressure variation, which did not respond to medical treatment, ocular massage, and anterior chamber paracentesis.

## 2. Patient and Methods

A 45-year-old male patient was presented to the clinic of ophthalmology with an advanced level of visual acuity loss that started suddenly in his right eye 1 hour ago. Informed consent was obtained from the patient for all treatment and surgical procedures. Complete ophthalmologic examination and fundus fluorescein angiography (FFA) were performed preoperatively and postoperatively.

Sublingual 10 mg isosorbide dinitrate (Isordil, Actavis, Turkey) and oral 100 mg acetylsalicylic acid (Coraspin, Bayer AG, Germany) were given to the patient, and ocular massage was performed to the eye immediately. Anterior chamber paracentesis was performed 2 hours later. Transconjunctival sutureless 25-gauge PPV (AA) was performed to the right eye. After core vitrectomy, the posterior hyaloid was detached with the help of triamcinolone acetonide (Kenacort, Deva, Turkey).

Intraoperative cycling pressure variation was induced in a five-second period with 650 mmHg venturi pump power in the closest position to the optic nerve head (Constellation, Alcon, USA). The same application was done at 15-second intervals. Intraocular pressure adjustment of the device was determined to be 35 mmHg during active suction and 15 mmHg between the intervals with continuing infusion. Intraoperative cycling pressure variation on the optic nerve head was repeated until blood flow in the central retinal artery was observed.

FFA imaging was performed again on the first day, postoperatively. Blood analysis, transesophageal echocardiography, and carotid artery Doppler ultrasonography were also performed for etiological evaluation. The patient was recommended to continue taking 100 mg of acetylsalicylic acid postoperatively. The patient was followed up for 1 month in the postoperative period.

## 3. Results

**Figure 1:**
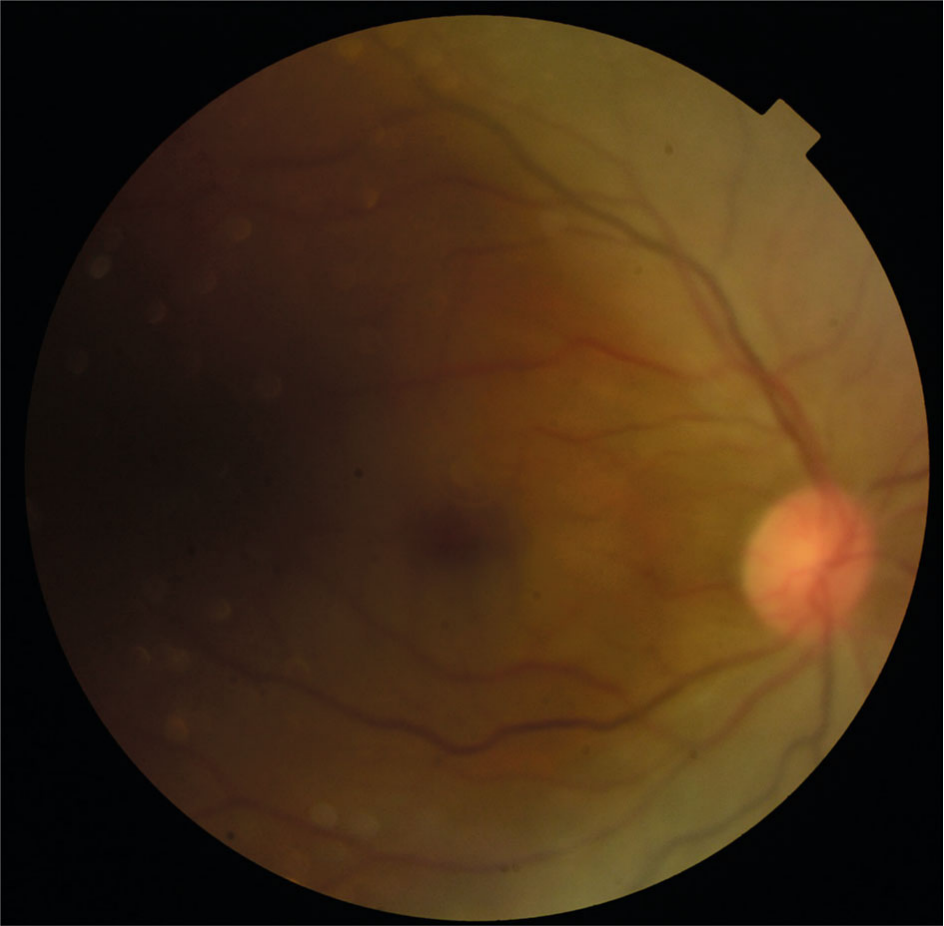
Retinal edema with arteriolar attenuation and cherry spot appearance in the right eye.

**Figure 2:**
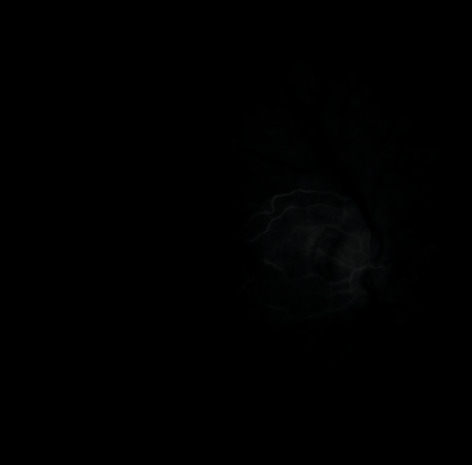
Preoperative low retinal perfusion in the right eye.

The best-corrected visual acuity (BCVA) was no light perception in the right eye and 20/20 in the left eye. There was relative afferent pupillary defect in the right eye. In biomicroscopic examination, anterior segment findings were within normal limits in both eyes. Funduscopic examination showed retinal edema with arteriolar attenuation and cherry spot appearance in the right eye (Figure 1). In FFA, retinal blood flow in the right eye was found to be very low (Figure 2). Funduscopic findings of the left eye were within normal limits. Intraocular pressures (IOP) were 15 mmHg and 16 mmHg in the right and left eyes, respectively.

**Figure 3:**
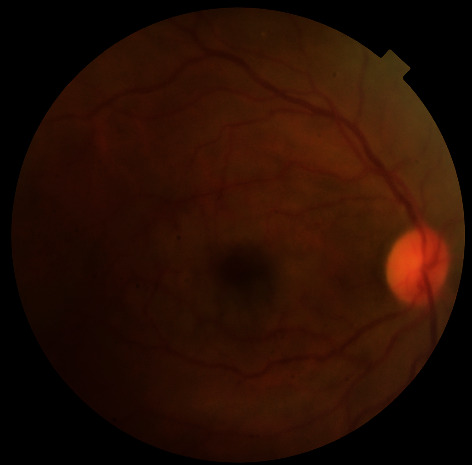
Postoperative fundus photograph of the right eye.

**Figure 4:**
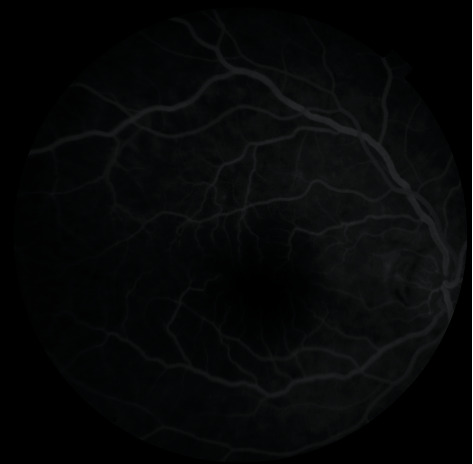
Postoperative retinal reperfusion of the right eye.

After ocular massage and anterior chamber paracentesis, there was no improvement in visual acuity and funduscopic findings. At the end of vitrectomy, there was no blood flow in the central retinal artery. In the eleventh active suction period, it was observed that the blood flow in the retinal artery started again, intraoperatively. On the first postoperative day, control FFA imaging revealed that edema and arterial attenuation in the retina disappeared (Figure 3) and retinal blood flow in the right eye was equally similar to the left eye (Figure 4). BCVA improved to 20/100 on the first day. During follow-up, BCVA in the right eye improved to 20/40 on the first week and to 20/20 on the first month, postoperatively.

All of the blood test results (partial thromboplastin time, activated partial thromboplastin time, international normalized ratio, and complete blood count) were within normal limits. Echocardiography showed no focus of thromboembolism in the heart. Only two atherosclerotic plaques were detected on the right side in Doppler ultrasonography of the internal carotid artery.

## 4. Discussion

Central retinal artery occlusion (CRAO) is an ophthalmic emergency that can cause significant and irreversible visual impairment. Although FFA is the gold standard in diagnosis, funduscopic examination findings (retinal edema, cherry red spot) and the presence of relative afferent pupillary defects are also helpful. Although it is more common in men, the incidence of CRAO is approximately 1 per 100,000 people [14]. The most common cause of occlusion is embolism, and the content of emboli could be cholesterol, calcium, and platelet-fibrin. The source of cholesterol and platelet-fibrin containing emboli is usually the heart or internal carotid artery [15]. In our case, there were two atherosclerotic in the right internal carotid artery.

Although experimental studies show that permanent damage in the retina develops to complete occlusion in 90 minutes, in the presence of incomplete occlusion, vision regain could be achieved after delays of 8 to 24 hours, and this period may be extended in cases where macular perfusion is maintained by the cilioretinal artery [14]. In our case, although the occlusion level was high, retinal perfusion was sustained, albeit very little. Although 3 hours have passed, the apparent vision regain may be related to ongoing perfusion, albeit weak.

Cycling pressure variation of the IOP may be successful in some cases in order to move or dislodge the thromboembolism. For this purpose, different methods have been defined in the literature. Ocular massage [3], anterior chamber paracentesis [11], intravenous mannitol or acetazolamide, and topical antiglaucoma drugs [4, 5] are the main alternatives. We initially applied ocular massage to the eye, since it was a noninvasive option. When there was no success with ocular massage, we performed anterior chamber paracentesis in the operating room.

Inhalation of 10% carbogen or sublingual isosorbide dinitrate could be applied to induce vasodilatation in the retinal artery. Isosorbide dinitrate is a preferred molecule in the treatment of angina pectoris and heart failure today. Nitrate-derived drugs cause relaxation in venous vessels rather than arteries. This may be the reason for their low effectiveness in CRAO. Pentoxifylline is a methylxanthine derivative that is called “a rheologic modifier” for its effects on increasing the deformability of red blood cells and used for the treatment of intermittent claudication. Pentoxifylline has been used in a limited number of cases in the treatment of CRAO but is not preferred today due to its low efficacy [16].

Another noninvasive method is Nd:YAG laser embolectomy/embolysis. In a meta-analysis that Man et al. conducted to investigate 13 cases in the literature where the Nd:YAG laser was applied for CRAO, they reported that there was visual improvement in most of the cases, the common complication was vitreous hemorrhage, and high pulse energy may be detrimental [12]. In our case, we did not prefer the Nd:YAG laser since the exact location of the embolus was not clear in funduscopic imaging or examination.

In cases when medical and noninvasive methods fail, operational interventions may be required [17, 18]. In a study of 13 cases with CRAO, Kadonosono et al. reported that they applied a tissue plasminogen activator into the retinal artery to achieve arterial cannulation after PPV and provided significant visual success [19]. Takata et al. also reported similar success in their two-case presentation [13]. Lin et al. reported that BCVA improved to 20/25 with vitrectomy and retinal artery massage in a patient that was unresponsive to medical treatment and had branch retinal artery occlusion for 5 days [20]. Lu et al. reported that they achieved success by retrieving embolus with microsurgical forceps in a case with branch retinal artery occlusion [21]. In a study of 10 patients with CRAO, Almeida et al. reported that they massaged the retinal artery in the optic nerve head with a “special probe” after PPV and achieved three or more lines of improvement in BCVA in six cases [22]. The reason that Lu et al. achieved limited visual success in their study may be due to traumatic neuropathy developing in the optic nerve head during massage. Nadal et al. reported that vitrectomy with intrasurgical control of ocular hypotony may be effective in the treatment of CRAO [23].

In our case, CRAO was resolved with PPV and intraoperative cycling pressure variation. To induce cycling pressure variation, we performed active suction from a point very close to the optic nerve head. This application may be unreasonable due to the hydrostatic pressure effect mechanism. To avoid optic nerve trauma, active suction could also be performed in the vitreous cavity away from the optic nerve head.

## 5. Conclusion

In cases with central retinal artery occlusion that does not respond to digital ocular massage and medical treatment, PPV and intraoperative cycling pressure variation on the optic nerve head could be a successful and nontraumatic method in providing reperfusion of the retina.
